# Seroepidemiology of Infection with *Toxoplasma gondii* in General Population in a Central Mexican City

**Published:** 2020

**Authors:** José Manuel SALAS-PACHECO, Angel Antonio VÉRTIZ-HERNÁNDEZ, Ada Agustina SANDOVAL-CARRILLO, Azahel de Jesús RANGEL-LÓPEZ, Elizabeth Irasema ANTUNA-SALCIDO, Sergio Manuel SALAS-PACHECO, Luis Francisco SÁNCHEZ-ANGUIANO, Edna Madai MÉNDEZ-HERNÁNDEZ, Jesús HERNÁNDEZ-TINOCO, Francisco Xavier CASTELLANOS-JUÁREZ, Osmel LA LLAVE-LEÓN, Cosme ALVARADO-ESQUIVEL

**Affiliations:** 1. Institute for Scientific Research “Dr. Roberto Rivera-Damm”, Juárez University of Durango State, Durango, Mexico; 2. Coordinación Académica Regional Altiplano de la Universidad Autónoma de San Luis Potosí, San Luis Potosí, Mexico; 3. Biomedical Research Laboratory, Faculty of Medicine and Nutrition, Juárez University of Durango State, Durango, Mexico

## Dear Editor-in-Chief

There is not previous report about the seroepidemiology of *Toxoplasma gondii* infection in the central Mexican state of San Luis Potosí. Therefore, we aimed to determine the prevalence of *T. gondii* infection and the sociodemographic, clinical, behavioral, and housing characteristics associated with this infection in the general population in Matehuala City, San Luis Potosí.

This cross-sectional study was performed from January to April 2018. In total, 636 subjects (mean age: 36.95±17.67 years; range: 18–91 years) were included. Anti-*T. gondii* IgG antibodies were detected using a commercially available enzyme immunoassay “*T. gondii* IgG” kit (Diagnostic Automation/Cortez Diagnostics, Inc., Woodland Hills, California. USA). All sera with seroreactivity to *T. gondii* IgG were further tested for anti-*T. gondii* IgM antibodies by a commercially available enzyme immunoassay “*T. gondii* IgM” kit (Diagnostic Automation/Cortez Diagnostics, Inc.).

This study was approved by the Institutional Ethical Committee of the General Hospital of the Health Services in Matehuala City, Mexico.

Of the 636 subjects studied, 54 (8.5%) were positive for anti-*T. gondii* IgG antibodies. Twenty-two (40.7%) of these subjects positive for anti-*T. gondii* IgG antibodies were also positive for anti-*T. gondii* IgM antibodies.

In a search in the medical literature about studies of seroprevalence in urban general population in Mexico, only one previous study was found. In that study, a 6.1% seroprevalence of *T. gondii* infection in general population in the northern Mexican city of Durango was found (1). This seroprevalence is comparable to the one found in our present study. On the other hand, in an international context, the seroprevalence found in the general population in Matehuala was lower than seroprevalences in the general populations reported in Portugal (22%) (2), Italy (24.4%) (3), Netherlands (26.0%) (4), and Iran (39.3%) (5). It is not clear why the seroprevalence of *T. gondii* infection in general population in Matehuala is lower than those reported in general populations in other countries. It is likely that differences in the characteristics of the populations studied and in the environment among countries may explain the differences in the rate of *T. gondii* infection.

In the current study, we searched for risk factors associated with *T. gondii* seropositivity. Logistic regression showed that *T. gondii* infection was positively associated with consumption of goat meat (OR = 1.81; 95% CI: 1.01–3.24; *P*=0.04), and negatively associated with ham consumption (OR = 0.44; 95% CI: 0.21–0.94; *P*=0.03). Other sociodemographic, clinical, behavioral, and housing characteristics were not associated with *T. gondii* infection. Consumption of goat meat has been associated with *T. gondii* seropositivity in Mexico. In a study of patients suffering from epilepsy in Durango City, a positive association between *T. gondii* seropositivity and consumption of goat meat was found (6). On the other hand, the negative association between *T. gondii* seropositivity and ham consumption found in this study suggests that ham consumption was not an important contributing factor for infection in the population studied.

In this first study about the epidemiology of *T. gondii* infection in San Luis Potosí, Mexico, we reported a low seroprevalence of this infection in the general population in Matehuala City. Consumption of goat meat was an important factor associated with *T. gondii* infection in the population studied. Results may help in the design of optimal preventive measures against *T. gondii* infection.
